# Clinical experience of the use of fibrinogen concentrate for massive postpartum hemorrhage: a retrospective case series study

**DOI:** 10.1007/s00540-023-03247-8

**Published:** 2023-08-24

**Authors:** Yoshiaki Ishida, Yoichiro Homma, Takeshi Murakoshi, Yoshie Toba

**Affiliations:** 1 Department of Anesthesiology, Seirei Hamamatsu General Hospital, Naka Ku, 2-12-12 Sumiyoshi, Hamamatsu, Shizuoka 430-8558 Japan; 2 Department of Critical Care Medicine, Tokyo Metropolitan Bokutoh Hospital, Sumida-Ku, Tokyo, Japan; 3 Department of General Internal Medicine, Seirei Hamamatsu General Hospital, Hamamatsu, Japan; 4 Department of Obstetrics and Gynecology, Maternal and Perinatal Care Center, Seirei Hamamatsu General Hospital, Hamamatsu, Japan

**Keywords:** Fibrinogen concentrate, Placental abruption, Postpartum hemorrhage

## To the Editor

In Japan, fibrinogen concentrate (FC) was approved for insurance coverage in 2021 for the treatment of hypofibrinogenemia associated with massive postpartum hemorrhage (PPH). This approval has the potential to significantly impact blood transfusion strategies for massive PPH. Knowledge of the application of FC is crucial for the development of these strategies. However, due to the rarity of massive PPH cases and lack of insurance coverage for FC before 2021, only a few clinical studies [1–3] have investigated its efficacy in Japan. In our hospital, with the written informed consent of patients or their families, we have been using FC clinically for massive PPH since 2016. Therefore, this retrospective case series study aimed to accumulate insight by summarizing our experience of administering FC for massive PPH.

The Institutional Review Board of Seirei Hamamatsu General Hospital, which is also the research ethics committee, approved this single-center retrospective study (approval number: 3905; April 20, 2022).

We enrolled pregnant women who delivered at our hospital after 22 gestational weeks and received hemostatic management including FC for massive PPH in the operating room from an anesthesiologist between January 1, 2016 and December 31, 2021. The exclusion criterion was women who were referred from local clinics or secondary hospitals due to hemorrhage of unknown extent.

We collected data on the patients’ demographic characteristics including pregnancy variables, and clinical characteristics including preoperative, intraoperative, and postoperative information from the electronic medical records. The definitions of the patients’ characteristics and perioperative findings are summarized in Supplementary Table 1. According to the Japanese Clinical Practice Guide 2022 for massive postpartum hemorrhage (PPH) [4], massive PPH in a pregnant woman is defined as a shock index (SI) ≥ 1.5, which estimates hemorrhage to be ≥ 2500 mL, an obstetrical disseminated intravascular coagulation (DIC) score ≥ 8, or fibrinogen levels < 150 mg/dL. Blood transfusion [red blood cell (RBC), fresh frozen plasma (FFP), platelet concentrate (PC), cryoprecipitate, and FC] is recommended to save the mother’s life in cases with massive PPH. In Japan, the “obstetrical DIC score” is used for the diagnosis of obstetrical DIC (Supplementary Fig. 1). This score encapsulates underlying prenatal diseases, clinical symptoms, and blood test results, and the full score is 44 points. Notably, when the obstetrical DIC score for underlying diseases and clinical symptoms is ≥ 8, a diagnosis of obstetrical DIC can be made (suspected) and therapy for DIC is supposed to be initiated, without waiting for the laboratory blood test results [4]. In this study, we calculated the obstetrical DIC score at the first FC administration.

Continuous variables were presented as the mean with standard deviation (SD) or the median with range, and graphical methods were used to confirm the normal distribution of the variables. Ordinal variables were presented as the median [range], and categorical variables as counts (%). These data would have no significance since the sample size was small (n < 10); hence, we did not perform further statistical analyses.

This study included nine patients, all of whom survived. Supplementary Table 2 summarizes the patients’ demographic characteristics, including pregnancy variables. Supplementary Tables 3 and 4 summarize the preoperative and intraoperative characteristics of hemostasis surgery, respectively. The median [range] FC dose was 3 [3–9] g. At FC administration, the median [range] intraoperative bleeding volume (BV) and pre/intraoperative BV were 1054 [0–9022] mL and 2594 [0–9022] mL, respectively. The median [range] obstetrical DIC score at FC administration was 11 [5–19], and all patients who underwent hysterectomy had obstetrical DIC scores ≥ 11. Supplementary Table 5 summarizes the postoperative characteristics of the transfusions and adverse events. The mean (SD) intra/postoperative transfusion volume was 5337 (2846) mL, and the average quantity of transfusion products administered was 16 units of RBC, 19 units of FFP, and 19 units of PC, whose ratio approximated 1:1:1. Patient 9 underwent hematoma removal surgery after hemostasis surgery and was transfused with 6 units of RBC, 8 units of FFP, and 10 units of PC. We classified patients into two groups based on whether or not they had undergone hysterectomy (Supplementary Table 6). The hysterectomy group appeared to be slightly older than the non-hysterectomy group, while the other baseline characteristics seemed largely similar between the two groups. At FC administration, the non-hysterectomy group seemed to have lower median obstetrical DIC scores (6.5 vs 16) and pre/intraoperative BV (712 vs 2792 mL) than the hysterectomy group. Similarly, intra/postoperative transfusion volumes appeared to be lower in the non-hysterectomy group (2950 vs 7246 mL). However, the average transfusion product ratio in both groups approximated 1:1:1. Hysterectomy was avoided in all three patients with placental abruption (PA). As no statistical analysis was performed due to the small sample size, these observations should not be considered as definitive comparative outcomes.

In this study, atonic bleeding was the most common cause of PPH (n = 7), followed by PA (n = 3) and amniotic fluid embolism (AFE) (n = 2). PA and AFE are representative examples of consumption coagulopathy associated with obstetrical DIC, as observed in five patients. Fibrinogen was consumed rapidly in large quantities in these patients; their minimum fibrinogen levels (which were unmeasurable in two patients, i.e., < 40 mg/dL) were lower than those in patients with non-consumption coagulopathy. Notably, hysterectomy was avoided in three patients with PA from among five patients with consumption coagulopathy. The pathomechanism of PA involves activation of the coagulation cascade via tissue factor (TF) disruption of trophoblasts or decidua integrity in the maternal circulation, leading to uncontrolled systemic thrombin generation and DIC [5]. In contrast, AFE is caused by a combined mechanism that entails activation of the coagulation cascade by the TF-rich amniotic fluid debris into the maternal circulation, and further activation of the coagulation cascade by the acute maternal systemic inflammatory response [5]. We believe that PA is a better indication for FC, owing to the absence of a systemic inflammatory response.

This study reported details about daily clinical practice at our institution; however, it has some limitations. First, our findings are only suitable for hypothesis generation due to the small sample size. Second, this case series study was a retrospective observational design, which lacked blinding or randomization. Nevertheless, our findings remain significant, as randomized controlled trials with sufficient sample sizes are not feasible in the rare and urgent milieu of massive PPH. According to the Japanese Clinical Practice Guide 2022 for massive PPH [4], the Japan Society of Obstetrics and Gynecology is proposing a system to register all cases of patients administered FC in the obstetric field in the future. Therefore, we anticipate a large-scale, retrospective study on this topic.

In conclusion, this study provides crucial insights into the use of FC for managing massive PPH in Japan. Although this study was limited by its small sample size, all patients treated with FC survived, supporting its potential as a valuable therapeutic tool for PPH. It is noteworthy that hysterectomy was avoided in three patients with PA; therefore, PA may be a better indication for FC among the other consumption coagulopathies.

### Supplementary Information

Below is the link to the electronic supplementary material.

Supplementary file1 (PPTX 44 KB)

Supplementary file2 (DOCX 52 KB)

## Data Availability

The datasets used and/or analyzed during the current study are available from the corresponding author on reasonable request.
