# Supplementary material for: Clinical experience of the use of fibrinogen concentrate for massive postpartum hemorrhage: a retrospective case series study
Source: J Anesth. 2023 Aug 24;37(5):820–2. doi: 10.1007/s00540-023-03247-8 (PMC10543518; doi:10.1007/s00540-023-03247-8)
Supplement: Supplementary file 1 — Supplementary file1 (PPTX 44 KB) [file 540_2023_3247_MOESM1_ESM.pptx]

## Slide 1
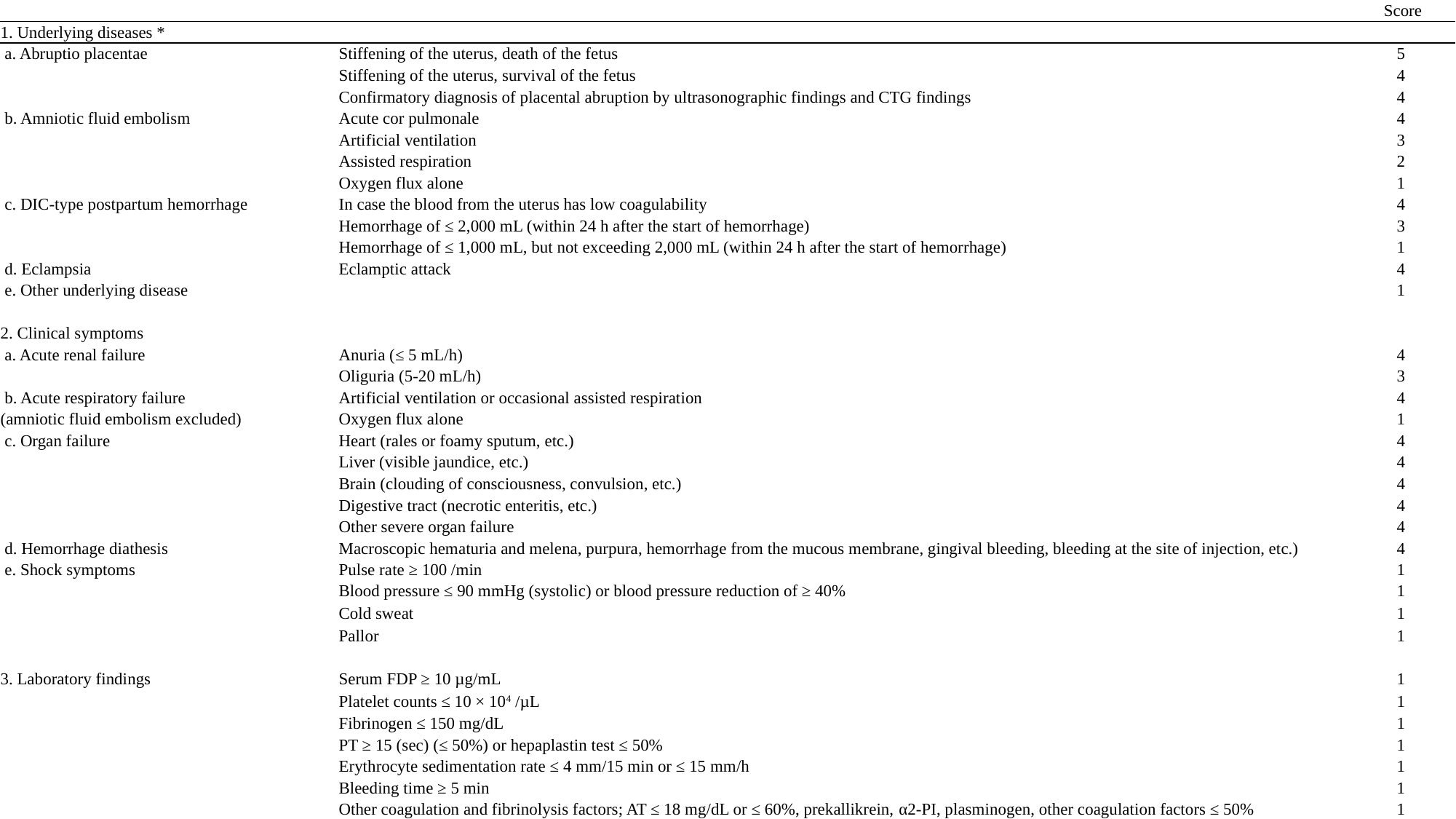

| | | Score |
| --- | --- | --- |
| 1. Underlying diseases \* | | |
| a. Abruptio placentae | Stiffening of the uterus, death of the fetus | 5 |
| | Stiffening of the uterus, survival of the fetus | 4 |
| | Confirmatory diagnosis of placental abruption by ultrasonographic findings and CTG findings | 4 |
| b. Amniotic fluid embolism | Acute cor pulmonale | 4 |
| | Artificial ventilation | 3 |
| | Assisted respiration | 2 |
| | Oxygen flux alone | 1 |
| c. DIC-type postpartum hemorrhage | In case the blood from the uterus has low coagulability | 4 |
| | Hemorrhage of ≤ 2,000 mL (within 24 h after the start of hemorrhage) | 3 |
| | Hemorrhage of ≤ 1,000 mL, but not exceeding 2,000 mL (within 24 h after the start of hemorrhage) | 1 |
| d. Eclampsia | Eclamptic attack | 4 |
| e. Other underlying disease | | 1 |
| | | |
| 2. Clinical symptoms | | |
| a. Acute renal failure | Anuria (≤ 5 mL/h) | 4 |
| | Oliguria (5-20 mL/h) | 3 |
| b. Acute respiratory failure | Artificial ventilation or occasional assisted respiration | 4 |
| (amniotic fluid embolism excluded) | Oxygen flux alone | 1 |
| c. Organ failure | Heart (rales or foamy sputum, etc.) | 4 |
| | Liver (visible jaundice, etc.) | 4 |
| | Brain (clouding of consciousness, convulsion, etc.) | 4 |
| | Digestive tract (necrotic enteritis, etc.) | 4 |
| | Other severe organ failure | 4 |
| d. Hemorrhage diathesis | Macroscopic hematuria and melena, purpura, hemorrhage from the mucous membrane, gingival bleeding, bleeding at the site of injection, etc.) | 4 |
| e. Shock symptoms | Pulse rate ≥ 100 /min | 1 |
| | Blood pressure ≤ 90 mmHg (systolic) or blood pressure reduction of ≥ 40% | 1 |
| | Cold sweat | 1 |
| | Pallor | 1 |
| | | |
| 3. Laboratory findings | Serum FDP ≥ 10 µg/mL | 1 |
| | Platelet counts ≤ 10 × 104 /µL | 1 |
| | Fibrinogen ≤ 150 mg/dL | 1 |
| | PT ≥ 15 (sec) (≤ 50%) or hepaplastin test ≤ 50% | 1 |
| | Erythrocyte sedimentation rate ≤ 4 mm/15 min or ≤ 15 mm/h | 1 |
| | Bleeding time ≥ 5 min | 1 |
| | Other coagulation and fibrinolysis factors; AT ≤ 18 mg/dL or ≤ 60%, prekallikrein, α2-PI, plasminogen, other coagulation factors ≤ 50% | 1 |
