# Supplementary material for: Clinical experience of the use of fibrinogen concentrate for massive postpartum hemorrhage: a retrospective case series study
Source: J Anesth. 2023 Aug 24;37(5):820–2. doi: 10.1007/s00540-023-03247-8 (PMC10543518; doi:10.1007/s00540-023-03247-8)
Supplement: Supplementary file 2 — Supplementary file2 (DOCX 52 KB) [file 540_2023_3247_MOESM2_ESM.docx]

**Electronic Supplementary Material**

**Supplementary Table 1.** Definitions of the patients’ characteristics and perioperative findings

| Data collection | Measurement variables and definitions |
| --- | --- |
| Physical characteristics | age, height, weight, and BMI |
| Pregnancy characteristics | gravidity, parity, number of pregnancies, obstetric comorbidities, mode of delivery, and gestational period |
| - Parity | included the current delivery, and the first birth was designated as para 1 |
| Preoperative characteristics for hemostasis surgery | use of CS and its indications, underlying pathophysiology of PPH requiring hemostasis surgery, anesthesia method, preoperative BV, and transfusion volume |
| - Underlying pathophysiology of PPH | included atonic bleeding, placenta previa, uterine inversion, uterine rupture, birth canal laceration and vaginal wall hematoma, PA, or AFE, which occurred singly or concomitantly |
| - Atonic bleeding | need for more than one uterotonic agent such as oxytocin, methylergometrine, and prostaglandin, because oxytocin is routinely used as an uterotonic agent in our hospital |
| - Preoperative BV | included BV at CS and persistent bleeding after vaginal delivery |
| Intraoperative characteristics | use of hysterectomy, FC dosage, lowest fibrinogen value, intraoperative BV, and transfusion volume |
| - Cesarean hysterectomy | hysterectomy performed during cesarean delivery |
| - Blood transfusion | transfusion of RBC, FFP, PC, Alb, and stored autologous blood |
| - Pre/intraoperative BV at FC administration | sum of preoperative and intraoperative BV at FC administration |
| - Pre/intraoperative BV at hysterectomy | sum of preoperative and intraoperative BV at hysterectomy |
| Postoperative characteristics | postoperative transfusion volume, adverse effects such as pulmonary edema and DVT, presence and duration of oxygen administration, ICU admission, mechanical ventilation, and dialysis |
| - Pulmonary edema | reduced radiographic translucency on chest X-ray that required oxygen and diuretic administration |
| - DVT | confirmed by lower limb vein echography |
| - Intra/postoperative transfusion volume | sum of the intraoperative and postoperative transfusion volumes |
| - Transfusion products | 2 units of RBC or FFP composed of 400 mL of whole blood, and 2 units of RBC, 2 units of FFP, and 10 units of PC correspond to 280 mL, 240 mL, and 200 mL, respectively, in Japan |

BMI: body mass index, CS: cesarean section, PPH: postpartum hemorrhage, BV: bleeding volume, PA: placental abruption, AFE: amniotic fluid embolism, FC: fibrinogen concentrate, RBC: red blood cells, FFP: fresh frozen plasma, PC: platelet concentrate, Alb: albumin products, DVT: deep vein thrombosis, ICU: intensive care unit,

**Supplementary Table 2.** Patients’ baseline characteristics

| Case | 1 | 2 | 3 | 4 | 5 | 6 | 7 | 8 | 9 |
| --- | --- | --- | --- | --- | --- | --- | --- | --- | --- |
| Year | 2016 | 2016 | 2018 | 2020 | 2020 | 2021 | 2021 | 2021 | 2021 |
| Age (years) | 37 | 36 | 40 | 33 | 29 | 38 | 39 | 35 | 32 |
| Height (cm) | 169 | 157 | 162 | 153 | 152 | 158 | 160 | 170 | 156 |
| Weight (kg) | 68.0 | 55.1 | 52.3 | 61.7 | 58.6 | 59.4 | 84.0 | 84.3 | 38.5 |
| BMI (kg/m^2^) | 23.8 | 22.4 | 19.9 | 26.4 | 25.4 | 23.8 | 32.8 | 29.2 | 19.9 |
| Gravidity | 1 | 5 | 2 | 2 | 1 | 3 | 3 | 11 | 1 |
| Parity | 0 | 2 | 1 | 1 | 0 | 2 | 2 | 4 | 0 |
| Gestational period (weeks) | 40 | 36 | 40 | 40 | 34 | 36 | 32 | 28 | 37 |
| Number of pregnancies | 1 | 1 | 1 | 1 | 1 | 1 | 1 | 1 | 1 |
| Obstetric comorbidities | HDP  Endometriosis | Total placenta previa  Previous CS | NA | NA | HDP | Total placenta previa  Previous CS | Polyhydramnios | HDP  Previous CS  Previous PA | NA |

BMI: body mass index, HDP: hypertensive disorders of pregnancy, CS: cesarean section, NA: not applicable, PA: placental abruption

**Supplementary Table 3.** Preoperative characteristics for hemostasis surgery

| Case | 1 | 2 | 3 | 4 | 5 | 6 | 7 | 8 | 9 |
| --- | --- | --- | --- | --- | --- | --- | --- | --- | --- |
| Presence of CS | Yes | Yes | No | No | Yes | Yes | No | Yes | Yes |
| Indications of CS | HDP | Total placenta previa | NA | NA | HDP  PA | Total placenta previa | NA | HDP  PA | PA |
| Underlying pathophysiology of PPH |  |  |  |  |  |  |  |  |  |
| Atonic bleeding | ◯ |  | ◯ | ◯ | ◯ | ◯ |  | ◯ | ◯ |
| Placenta previa |  | ◯ |  |  |  | ◯ |  |  |  |
| Uterine inversion |  |  | ◯ | ◯ |  |  |  |  |  |
| Uterine rupture |  |  |  |  |  |  |  |  |  |
| Birth canal laceration and vaginal wall hematoma |  |  | ◯ | ◯ |  |  |  |  |  |
| Placental abruption |  |  |  |  | ◯ |  |  | ◯ | ◯ |
| Amniotic fluid embolism | ◯ |  |  |  |  |  | ◯ |  |  |
| Anesthesia method | GA | CSEA to GA | GA | GA | GA | CSEA to GA | GA | SA | GA with PNB |
| Preoperative BV (mL) | 2390 | 0 | 1594 | 2336 | 0 | 0 | 1292 | 0 | 0 |

CS: cesarean section, HDP: hypertensive disorders of pregnancy, NA: not applicable, PA: placental abruption, PPH: postpartum hemorrhage, GA: general anesthesia, CSEA: combined spinal-epidural anesthesia, SA: spinal anesthesia, PNB: peripheral nerve block, BV: bleeding volume

**Supplementary Table 4.** Intraoperative characteristics for hemostasis surgery

| Case | 1 | 2 | 3 | 4 | 5 | 6 | 7 | 8 | 9 |
| --- | --- | --- | --- | --- | --- | --- | --- | --- | --- |
| Hysterectomy | Yes | Yes | Yes | No | No | Yes | Yes | No | No |
| Cesarean hysterectomy | Yes | Yes | No | No | No | Yes | No | No | No |
| Minimum fibrinogen levels (mg/dL) | 58 | 93 | 121 | 174 | 58 | 113 | <40 | <40 | 87 |
| FC dosage (g) | 3 | 3 | 3 | 3 | 6 | 3 | 9 | 6 | 6 |
| DIC score at FC administration | 19 | 16 | 14 | 5 | 11 | 11 | 19 | 7 | 6 |
| Intraoperative BV at FC administration (mL) | 0 | 9022 | 1000 | 1309 | 0 | 5136 | 1500 | 1054 | 370 |
| Pre/intraoperative BV at FC administration (mL) | 2390 | 9022 | 2594 | 3645 | 0 | 5136 | 2792 | 1054 | 370 |
| Intraoperative BV at hysterectomy (mL) | 3780 | 9022 | 1000 | NA | NA | 5136 | 3000 | NA | NA |
| Pre/intraoperative BV at hysterectomy (mL) | 6170 | 9022 | 2594 | NA | NA | 5136 | 4292 | NA | NA |
| Intraoperative BV (mL) | 3880 | 14377 | 1440 | 1309 | 1590 | 5415 | 3490 | 1054 | 370 |
| Intraoperative transfusion volume (mL) | 6710 | 10350 | 4160 | 1460 | 1660 | 4920 | 4520 | 480 | 1660 |
| RBC (mL) | 2380 | 4990 | 2320 | 980 | 560 | 1680 | 1680 | 0 | 740 |
| FFP (mL) | 3480 | 3600 | 1240 | 480 | 700 | 1440 | 2640 | 480 | 920 |
| PC (mL) | 600 | 300 | 600 | 0 | 400 | 0 | 200 | 0 | 0 |
| Alb (mL) | 250 | 500 | 0 | 0 | 0 | 500 | 0 | 0 | 0 |
| Stored autologous blood (mL) | 0 | 960 | 0 | 0 | 0 | 1300 | 0 | 0 | 0 |

FC: fibrinogen concentrate, DIC: disseminated intravascular coagulation, BV: bleeding volume, NA: not applicable, RBC: red blood cells, FFP: fresh frozen plasma, PC: platelet concentrate, Alb: albumin products

**Supplementary Table 5.** Postoperative characteristics: Transfusions and adverse events

| Case | 1 | 2 | 3 | 4 | 5 | 6 | 7 | 8 | 9 |
| --- | --- | --- | --- | --- | --- | --- | --- | --- | --- |
| Transfusions | | | | | | | | | |
| Postoperative transfusion volume (mL) | 740 | 1430 | 920 | 380 | 820 | 1240 | 1240 | 2760 | 2580 |
| RBC (mL) | 420 | 610 | 480 | 140 | 560 | 560 | 560 | 1120 | 940 |
| FFP (mL) | 120 | 720 | 440 | 240 | 260 | 480 | 480 | 1440 | 1240 |
| PC (mL) | 200 | 100 | 0 | 0 | 0 | 200 | 200 | 200 | 400 |
| Intra/postoperative transfusion volume (mL) | 7450 | 11780 | 5080 | 1840 | 2480 | 6160 | 5760 | 2340 | 4240 |
| Intra/postoperative RBC (units) | 20 | 40 | 20 | 8 | 8 | 16 | 16 | 8 | 12 |
| Intra/postoperative FFP (units) | 30 | 36 | 14 | 6 | 8 | 16 | 26 | 16 | 18 |
| Intra/postoperative PC (units) | 40 | 20 | 30 | 0 | 20 | 10 | 20 | 10 | 20 |
| Adverse events | | | | | | | | | |
| Pulmonary edema | Yes | No | No | No | Yes | No | Yes | No | No |
| DVT | No | No | NA | NA | No | NA | NA | NA | NA |
| Duration of oxygen administration (days) | 1 | 1 | 1 | 1 | 1 | 0 | 1 | 0 | 0 |
| Return destination | ICU | ICU | ICU | MFICU | MFICU | MFICU | ICU | MFICU | MFICU |
| Duration of ICU stays (days) | 3 | 3 | 2 | 0 | 0 | 0 | 2 | 0 | 0 |
| Duration of mechanical ventilation (days) | 2 | 2 | 0 | 0 | 0 | 0 | 2 | 0 | 0 |
| Dialysis | No | No | No | No | No | No | No | No | No |

RBC: red blood cells, FFP: fresh frozen plasma, PC: platelet concentrate, ICU: intensive care unit, MFICU: maternal fetal intensive care unit, NA: not applicable, DVT: deep vein thrombosis

**Supplementary Table 6.** Patient characteristics with respect to hysterectomy

|  | Hysterectomy (n = 5) | Non-hysterectomy (n =4) | Total (n = 9) |
| --- | --- | --- | --- |
| Age (years) | 38.0 (1.4) | 32.3 (2.2) | 35.4 (3.4) |
| Height (cm) | 161 (4.3) | 158 (7.2) | 160 (6.0) |
| Weight (kg) | 63.8 (11.4) | 63.3 (13.1) | 63.5 (12.2) |
| BMI (kg/m^2^) | 24.5 (4.4) | 25.2 (3.4) | 24.8 (4.0) |
| Gravidity | 3 [1–5] | 1.5 [1–11] | 2 [1–11] |
| Parity | 2 [0–2] | 0.5 [0–4] | 1 [0–4] |
| Gestational period (weeks) | 36.8 (3.0) | 34.8 (4.4) | 35.9 (3.8) |
| Underlying pathophysiology of PPH |  |  |  |
| Atonic bleeding | 3 (60%) | 4 (100%) | 7 |
| Placenta previa | 2 (40%) | 0 (0%) | 2 |
| Uterine inversion | 1 (20%) | 1 (25%) | 2 |
| Uterine rupture | 0 (0%) | 0 (0%) | 0 |
| Birth canal laceration and vaginal wall hematoma | 1 (20%) | 1 (25%) | 2 |
| Placental abruption | 0 (0%) | 3 (100%) | 3 |
| Amniotic fluid embolism | 2 (40%) | 0 (0%) | 2 |
| FC dosage (g) | 3 [3–9] | 6 [3–6] | 3 [3–9] |
| DIC score at FC administration | 16 [11–19] | 6.5 [5–11] | 11 [5–19] |
| Preoperative BV (mL) | 1292 [0–2390] | 0 [0–2336] | 0 [0–2390] |
| Intraoperative BV at FC administration (mL) | 1500 [0–9022] | 712 [0–1309] | 1054 [0–9022] |
| Pre/intraoperative BV at FC administration (mL) | 2792 [2390–9022] | 712 [0–3645] | 2594 [0–9022] |
| Intraoperative BV (mL) | 3880 [1440–14377] | 1181.5 [370–1590] | 1590 [370–14377] |
| Intraoperative transfusion volume (mL) | 6132 (2284) | 1315 (489) | 3991 (2955) |
| Postoperative transfusion volume (mL) | 1114 (247) | 1635 (1049) | 1346 (768) |
| Intra/postoperative transfusion volume (mL) | 7246 (2395) | 2950 (895) | 5337 (2846) |
| Intra/postoperative RBC (units) | 22.4 (9.0) | 9.0 (1.7) | 16 (10.0) |
| Intra/postoperative FFP (units) | 24.4 (8.3) | 12.0 (5.0) | 19 (9.0) |
| Intra/postoperative PC (units) | 24.0 (10.1) | 12.5 (8.3) | 19 (11.0) |
| Pulmonary edema | 2 (40%) | 1 (25%) | 3 |

Values are presented as means (SD), median [range], or counts (%).

BMI: body mass index, PPH: postpartum hemorrhage, FC: fibrinogen concentrate, DIC: disseminated intravascular coagulation, BV: bleeding volume, RBC: red blood cells, FFP: fresh frozen plasma, PC: platelet concentrate

**Supplementary Figure 1.** Obstetrical DIC score

DIC: disseminated intravascular coagulation

A diagnosis of obstetrical DIC can be made when the obstetrical DIC score is ≥ 8, and therapy for DIC is supposed to be initiated, without waiting for the coagulation test results. An obstetrical DIC score >13, including at least two coagulation tests, is compatible with the diagnostic criteria for DIC.

* Only one of these items will be adopted as the score for underlying diseases.
